# Comparative analysis of skin microbiome of patients with filarial lymphedema and healthy individuals

**DOI:** 10.1371/journal.pone.0325380

**Published:** 2025-07-02

**Authors:** Sowmiya Manavalan, Devu Pradeep, Danapriyaa Dharmalingam, Janani Semalaiyappan, Thamizhpraba Sivarasan, Shakila Venkatesan, Sankari Thirumal, Vijesh Sreedhar Kuttiatt

**Affiliations:** Unit of Clinical and Molecular Medicine, ICMR-Vector Control Research Centre (VCRC), Puducherry, India; Rani Lakshmi Bai Central Agricultural University, INDIA

## Abstract

**Background:**

Lymphatic filariasis, a vector borne parasitic disease is a public health problem in the tropical region. Recurrent skin and soft tissue infections termed adenolymphangitis (ADL) is a major complication of filarial lymphedema. Understanding the changes in skin microbiome associated with this disease may provide novel insights on ADL attacks and lymphedema progression. This study investigates the changes in skin microbial flora in patients affected with filarial lymphedema.

**Methods:**

We employed 16S rRNA gene amplicon-based metagenomic technique to profile the skin microbiome of patients with filarial lymphedema in comparison with healthy volunteers.

**Results:**

There were notable differences in the bacterial flora between patients and healthy controls. Actinobacteria were under-represented in the patient group. *Staphylococcus* dominated both the groups, 63% in patients and 44% in controls. Samples from a few patients showed the presence of certain rare bacteria like *Eremococcus* and *Facklamia.*

**Conclusion:**

This pilot study applying advanced molecular tools provides insight on the changes in skin microflora associated with filarial lymphedema for the first time. Further studies are necessary for a better understanding of the role of the altered skin microbiome in frequent episodes of adenolymphangitis in patients with filarial lymphedema.

## Introduction

Lymphatic filariasis (LF) is a tropical vector-borne parasitic disease caused by the filarial worms *Wuchereria bancrofti, Brugia timori and Brugia malayi*, *W. bancrofti* being the most prevalent one. These worms invade human lymphatics and induce pathological changes that subsequently manifest as limb lymphoedema or hydrocele. This illness, the second leading cause of physical disability, affects an estimated 120 million individuals across 83 countries [1]. Lymphedema of the limbs is the most common manifestation of LF. Hydrocele and breast enlargement are also known manifestations. [2]. In 2000, the World Health Organisation (WHO) initiated the Global Programme for Elimination of LF (GPELF). Two primary components of this are (a) the annual mass drug administration (MDA) program to stop the spread of infection and (b) the morbidity management and disability prevention (MMDP) initiative to lessen disability caused by the illness, which is focused on home-based limb care [3].

Lymphedema predisposes to recurrent skin and soft tissue infections termed ADL (Adenolymphangitis) attacks. Disease-related alteration in the skin microbiome may play a major role in frequent ADL attacks as skin acts as the portal of entry for bacteria, especially *Streptococcus and Staphylococcus* [4,5]*.* Previous studies reported the association between group A streptococcal infection and ADL attacks in LF patients, as evidenced by the significant increase in anti-streptolysin O (ASO) titres [6]. Recent studies indicate that skin infections with *Staphylococci* and gram-negative bacteria are also common in patients with filarial lymphedema [7]. Maintenance of limb hygiene is the single most important factor that protects the patients from recurrent ADL attacks, which points towards a significant role of the skin microflora in these patients.

Lately, there has been a great interest in understanding the microbiome in different body systems, including skin, and their role in different disease conditions. Studies on the skin microbiome in dermatological conditions like psoriasis, atopic dermatitis, and acne indicate differences between patients and healthy individuals. To our knowledge, there is scant data on skin microbiome in patients with filarial lymphedema [8]. As lymphedema progresses, skin barrier dysfunction and persistent inflammation create an environment for an altered skin microbiome. Focusing on these advanced stages will reveal the microbial alterations, particularly with secondary infections, chronic inflammation, and potential disease complications. Significant changes in the microbiome of grade III and IV patients compared to healthy individuals, could indicate that microbial dysbiosis worsens as the disease progresses. We explored the skin microbiome of filarial lymphedema compared to that of healthy controls, employing 16S rRNA gene-based metagenomics. This high throughput technique has the additional advantage of identifying unculturable and difficult-to-culture organisms as well [9].

While existing research, such as Kwarteng et al. (2022), has begun to explore shifts in the skin microbiome in filarial lymphedema, and Campbell et al. (2023) has contributed valuable insights in secondary lymphedema, the overall data landscape remains remarkably sparse, particularly concerning LF patient populations [10,11]. This scarcity of data hinders our ability to fully appreciate the role of the skin microbiome in health and disease. Our work is significant because it employs a high-throughput approach to comprehensively characterize the differences in the skin microbiome between patients and controls. Our work leverages a high-throughput approach to comprehensively characterize the skin microbiome, enabling a robust comparison between patients and controls. This high-throughput methodology allows for a more in-depth analysis of the microbial community structure and function than traditional methods, potentially revealing subtle but significant differences that might be missed with less sensitive techniques. This detailed characterization is crucial for identifying specific microbial signatures associated with patient status, ultimately paving the way for improved diagnostics and personalized treatment strategies.

## Methodology

The aim of this study is to understand the skin microbial composition and diversity in patients with filarial lymphedema and compare it with healthy individuals. The objectives include: (1) to identify bacterial ecology associated with filarial lymphedema using 16S rRNA gene amplicon based metagenomic sequencing with culture-based methods, (2) assessing differences in microbiome composition compared to other inflammatory skin diseases in previously published works.

### Study setting

This project was conducted at the Filariasis Management Clinic of ICMR-VCRC. The study participants included patients with filarial lymphedema grade III or grade IV (n = 10) based on Four-Grade classification of filarial lymphedema. The grade III and grade IV filarial lymphedema patients were included to understand microbial shifts associated with advanced disease stages (skin folds, knobs etc) [2] in comparison to apparently, healthy individuals (n = 10) who were staff of VCRC. Participants with skin diseases, diabetes, and immunodeficiency were excluded. The study was carried out during a period of 6 month from 02^nd^ February 2023–31^st^ July 2023. Ethical approval was obtained from the Institutional Human Ethics Committee of VCRC (IHEC-0222/N/F) and written informed consent was obtained from the participants.

### Sample collection

Swab samples were collected from the medial aspect of the affected leg of the patients and the right leg of healthy individuals employing the previously described method [12]. Sterile cotton swabs soaked in NaCl (Himedia Laboratories Pvt. Ltd, Mumbai, India) were used to collect the samples. Once collected, the samples were stored in nutrient broth at −80°C until processing.

### Bacterial culture

Collected skin swab samples were cultured in a nutrient broth medium overnight. For this, the swab sponge was thoroughly swirled and pressed against the tube wall multiple times to ensure the bacteria were transferred into the nutrient solution. 250 μl of this solution was added to 10 ml of freshly prepared, sterilised nutrient broth in a tube and incubated overnight. From this, 500 μl culture inoculum was transferred into a 1.5 ml microcentrifuge tube and taken for extraction of DNA.

### DNA isolation and next-generation sequencing

DNA isolation was carried out using Sigma-Aldrich kit (Sigma Aldrich Co. LLC Missouri, USA) as per the manufacturer’s instruction. DNA content and purity were measured using Multiskan GO spectrophotometer (Thermofisher Scientific Oy Ratastie, Finland) with absorbance at 260/280 nm. The V3-V4 hypervariable region of the 16S rRNA gene was amplified using specific primers - 341F (5’-CCT AYG GGR BGC ASC AG-3’) and 806R (5’-GGA CTA CNN GGG TAT CTA AT-3’). Phusion® High-Fidelity (Thermofisher Scientific Oy Ratastie, Finland) PCR master mix was used for the PCR experiments. The PCR conditions were initial denaturation at 95°C for 3 min, followed by 40 cycles of denaturation at 94°C for 1 min, annealing at 57°C for 30 sec, elongation at 72°C for 1 min, and holding at 4°C for 3 minutes. PCR products were mixed with an equal volume of 1X loading buffer and electrophoresed on a 2% agarose gel. PCR products were purified using a Gel purification kit (Qiagen, Germany). Purified PCR products were subjected to library preparation and generated with an Illumina DNA preparation kit, and the final purified amplicons were quantified using Qubit. The DNA concentrations were adjusted to facilitate high-throughput sequencing using the Illumina NovaSeq 6000 platform to generate 250-bp paired-end reads. Next generation sequencing was outsourced to a sequencing firm.

### Bioinformatics analysis

Raw sequence reads were converted into FASTQ files after de-multiplexing it based on the Illumina index reads. The quality of the raw reads has been evaluated using the FastQC tool [13]. DNA reads were assembled into contigs, and BLAST search was performed to compare the contigs against the 16S rRNA reference database during the pre-processing stage. The entire set of raw sequencing reads was analysed for characteristics like GC distribution, base quality, and base composition. Several filters, including mismatch, conserved region, and spacer were used to eliminate low-quality reads. After eliminating low-quality reads, cleaned reads were analysed using the QIIME2 (version 2022.2) [14]. Sequence alignment was performed with the SILVA and Green Genes RNA databases as references. The USEARCH and UCLUST algorithms were used for chimera filtering, and operational taxonomic units (OTU) were grouped at 97% sequence identity threshold for taxonomic assignment [14]. The sequence was denoised via Deblur, which will merge and denoise the paired-end reads and produce the feature table and representative sequences. In the Deblur pipeline, quality filtering was performed before chimera checking, ensuring that only high-quality reads were used before identifying and removing chimeric sequences. The data were processed with the Krona tool for metagenomic visualization to display the relative abundance of microbiota. The microbial composition of patients and controls was shown at the individual and group levels using Krona plots (psadd software, version 0.1.3) [15]. The rarefaction curve (Vegan software, version 2.6–8) was used to measure the richness of the microbiota since it displayed patterns that were similar between biological replicates.

### Microbial community structure and diversity analysis

Diversity analysis was performed using the software QIIME2 (version 2022.2) and R package qiime2R (version 0.99.6). Rarefaction curves were generated to understand sequencing depth across samples. The samples were normalized before downstream analysis. Rarefied OTU table containing OTU counts were used for diversity calculations across samples. Alpha diversity is used to analyse the complexity of species diversity for all samples through several indices including estimate evenness, species richness, observed species, Chao1, Shannon, and Simpson indices [16,17]. The Shannon Index estimates species diversity, while Chao1-based analysis represents OTU richness. The rank abundance curve demonstrates the species richness and evenness [18]. Beta diversity analysis was performed to evaluating the species complexity using Vegan software (version 2.6–8). It was measured using various indices which include Pearson, weighted UniFrac, unweighted UniFrac, and Bray–Curtis. Principal component analysis (PCA) was carried out based on both weighted and unweighted Unifrac distances to compare the microbial communities between the groups and visualize the results in a two-dimensional coordinate graph to reflect variation among patient and control groups. Further visualization was done using the qiime2R (version 0.99.6) and phyloseq 1.48.0 R packages.

MAFFT software was used to align the representative sequences to interpret the phylogenetic relationship of all OTUs [19]. The sites that are not phylogenetically informative were masked, and the phylogenetic tree was constructed. Representative sequences for each OTU were screened for further annotation. The pre-trained Naive Bayesian classifier (SILVA database) was used to classify each read or amplicon sequence variant (ASV).

## Results

Twenty participants were enrolled in the study, 10 patients with filarial lymphedema and 10 apparently healthy individuals. The demographic and clinical characteristics of the patients and controls are summarized in Table 1. All patients were suffering from advanced disease, grade III or IV lymphedema. None of the participants had diabetes or other skin disorders, and none had received antibiotics in the past month before recruitment in the study. 16S rRNA amplicon sequencing-based analysis was carried out in 10 patients and 9 healthy controls. Of the 10 control sequences, one healthy control participant (MN) was excluded from the analysis due to low read count (178 reads) compared to the others (>120,000 reads). This outlier was excluded to prevent potential bias in downstream analyses.

**Table 1:** Characteristics of patients and controls.

Patients						Controls			
Sl.No	Patient ID	Age (Yrs)	Sex	Lymphedema grade	Duration of lymphedema (Yrs)	Sl. No	Participant ID	Age (Yrs)	Sex
1	KS	55	M	IV	4	1	MJ	58	M
2	AR	46	M	IV	5	2	BN	59	M
3	RN	65	M	IV	9	3	SN	57	M
4	BR	58	M	III	6	4	GR	52	M
5	JL	73	M	III	7	5	#MN	56	M
6	GN	50	M	IV	5	6	RM	62	F
7	PN	62	M	IV	6	7	ID	50	F
8	JM	60	M	IV	8	8	IN	50	F
9	RA	53	F	IV	11	9	MK	60	F
10	KN	63	F	IV	7	10	GT	58	F

# poor quality sequence reads and hence excluded from analysis.

### Microbial community and their taxonomic distribution

Bacteria detected in both patient and control groups belonged mainly to the phyla Firmicutes, Proteobacteria, and Actinobacteria*.* Overall, 24 genera were identified. However, some of the sequences could not be identified, from both patients and controls. The common genera detected in all samples include *Staphylococcus and Bacillus*. Importantly, certain genera, namely *Aerococcus, Facklamia, Eremococcus, Enhydrobacter, Helcococcus, Idiomarina, Macrococcus, Terribacillus, Dermabacter, Arcanobacterium and Mannheimia* were detected in some patients, but not in any of the samples from controls. On the other hand, *Paenibacillus* was present in one control sample but in none of the samples from patients.

### Bacterial flora in patients

Bacteria detected in patients mainly belonged to the phylum Firmicutes followed by Proteobacteria and Actinobacteria (Fig 1). The class Bacilli was the most prevalent within Firmicutes, followed by Gammaproteobacteria and Actinobacteria, with Pseudomonadales as the dominant order. Families Bacillales, Paenibacillaceae, Enterococcaceae, Aerococcaceae, and Streptococcaceae were highly abundant. Dominant genera included *Staphylococcus (63%), Bacillus (15%), Aneurinibacillus (9%)*, *Corynebacterium (6%), Acinetobacter (1%), Enhydrobacter (1%), Moraxella (1%), Pseudomonas (1%), and Mannheimia (1%)*. Certain genera *Aerococcus, Facklamia*, *Eremococcus, Enhydrobacter, Helcococcus, Idiomarina, Macrococcus, Terribacillus, Dermabacter, Arcanobacterium and Mannheimia* were detected in some patients (Fig 1). The bacterial flora detected in each patient is summarised in Table 2.

**Table 2:** Bacterial genera in patients and control samples.

Patient ID	Bacterial flora	Healthy Control ID	Bacterial flora
KS	*Staphylococcus, Bacillus, Corynebacterium, Dermabacter, Terribacillus, Eremococcus, Facklamia, Enterococcus, Streptococcus*	MJ	*Staphylococcus, Bacillus*
AR	*Staphylococcus, Bacillus, Moraxella*	BN	*Staphylococcus, Bacillus, Paenibacillus*
RN	*Staphylococcus, Bacillus, Lysinibacillus, Pseudomonas*	SN	*Staphylococcus, Bacillus, Moraxella*
BR	*Staphylococcus, Bacillus, Corynebacterium, Dermabacter, Moraxella, Macrococcus, Streptomyces, Eremococcus*	GR	*Staphylococcus, Bacillus, Moraxella, Aneurinibacillus*
JL	*Corynebacterium, Moraxella, Streptomyces, Acinetobacter, Enhydrobacter*	#MN	*-- -- -- -- -- -- --*
GN	*Staphylococcus, Bacillus, Corynebacterium, Dermabacter, Acinetobacter, Enhydrobacter, Facklamia, Macrococcus, Eremococcus*	RM	*Staphylococcus, Bacillus, Enterococcus, Lysinibacillus, Moraxella*
PN	*Staphylococcus, Bacillus, Corynebacterium, Moraxella, Aerococcus, Macrococcus, Streptomyces, Idiomarina, Pseudomonas, Enhydrobacter*	ID	*Staphylococcus, Bacillus, Aneurinibacillus, Streptomyces*
JM	*Staphylococcus, Bacillus, Macrococcus, Terribacillus, Aneurinibacillus*	IN	*Staphylococcus, Moraxella, Acinetobacter*
RA	*Staphylococcus, Bacillus, Corynebacterium, Moraxella, Acinetobacter, Mannheimia, Streptococcus, Arcanobacterium, Acinetobacter, Enterococcus, Macrococcus, Helcococcus, Enhydrobacter, Eremococcus*	MK	*Staphylococcus, Bacillus, Streptococcus, Acinetobacter*
KN	*Staphylococcus, Bacillus*	GT	*Staphylococcus, Bacillus*

# poor quality sequence reads and hence excluded from analysis.

**Fig 1:**
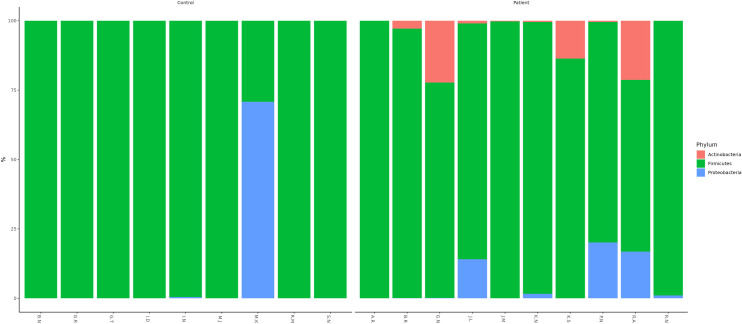
Bar plots representing the relative taxa abundance of each individual at the phylum level.

### Bacterial flora in healthy controls

Firmicutes were the most common phylum in the control group, followed by Proteobacteria (Fig 1). Within Firmicutes, the class Bacilli was abundant, and the order Bacillales was the dominant. The families Paenibacillaceae, Bacillaceae, and Staphylococcaceae were the most abundant ones. Dominant genera included *Staphylococcus (44%) Bacillus (31%), and Aneurinibacillus (12%).* Genus *Enterococcus (4%)* belonging to the order Lactobacillales was also noted. The bacterial flora detected in each healthy control are mentioned in Table 2.

**Fig 2:**
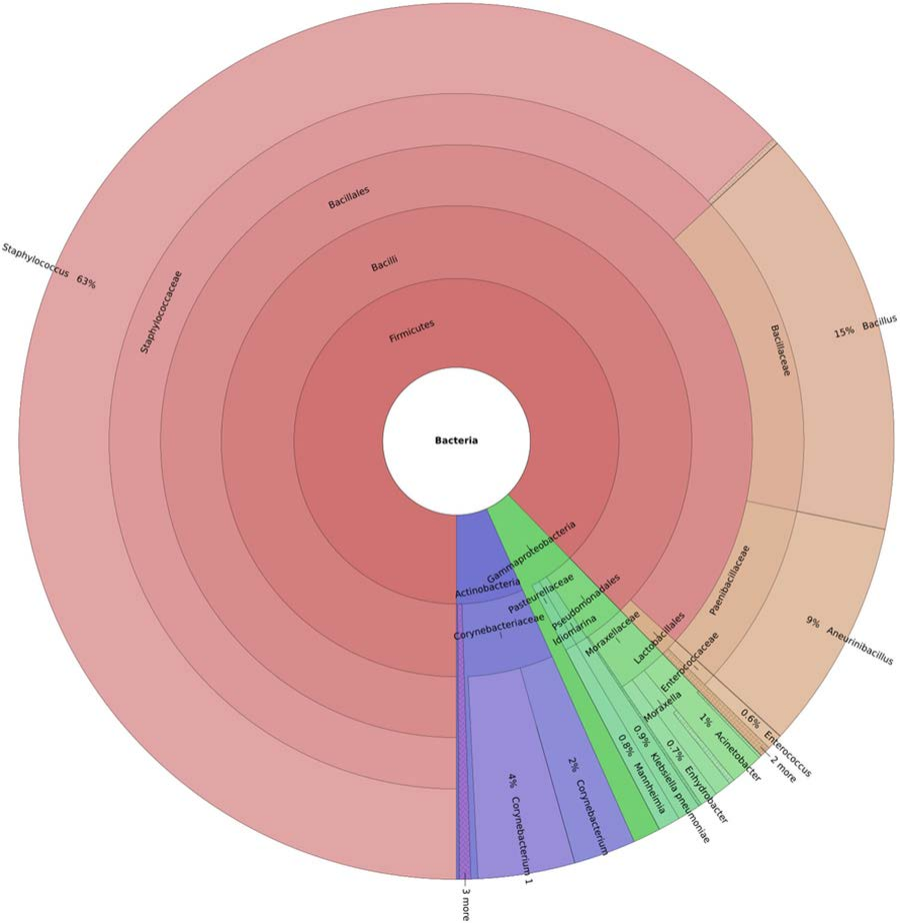
Krona plot representing relative taxa abundance in the patient group.

**Fig 3:**
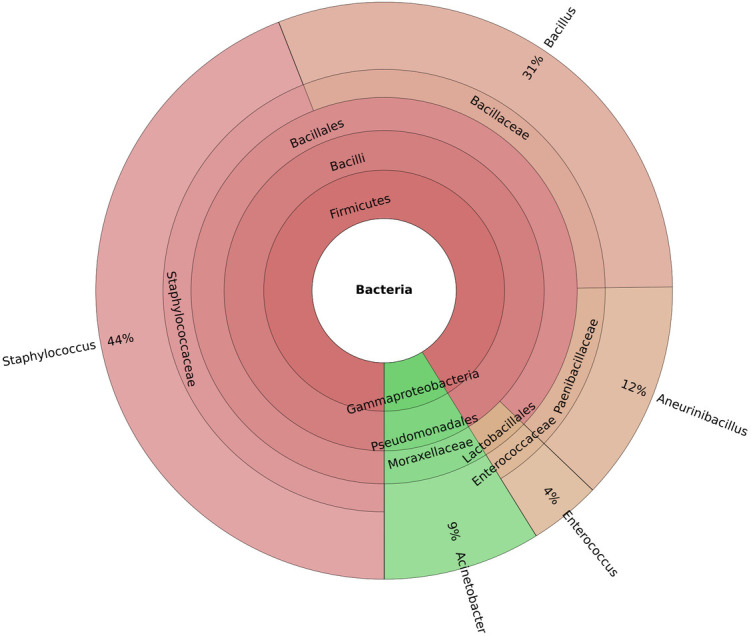
Krona plot representing relative taxa abundance in the control group.

**Fig 4:**
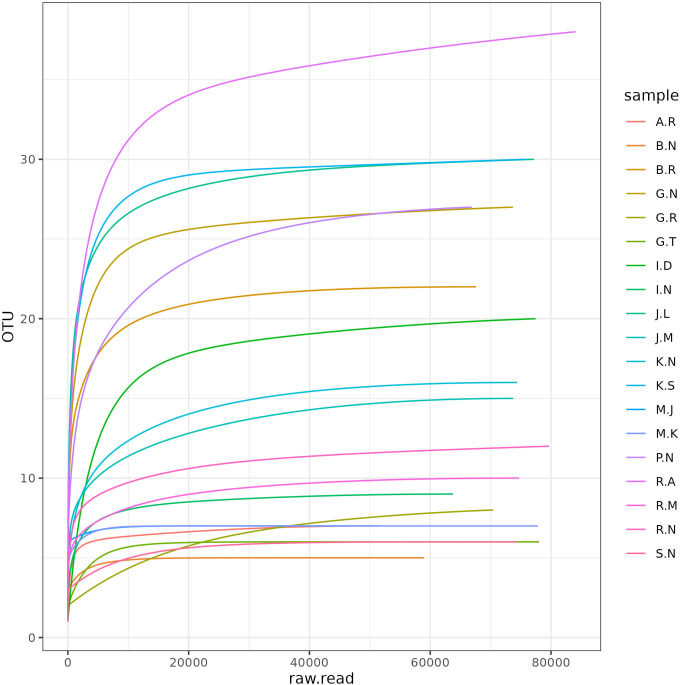
Rarefaction curve of the 16S rRNA Illumina libraries in the patient and control group.

Figs 2 and 3 represent the Krona plots displaying *Staphylococcus* as the most abundant genus, 63% in the patient group and 44% in the control group. The heat tree (S1 Fig) and the heat map (S2 Fig) depict the same. The Rarefaction curves were calculated for each sample, depicting the microbial community diversity in both patient and control groups (Fig 4).

### Diversity analysis

**Fig 5:**
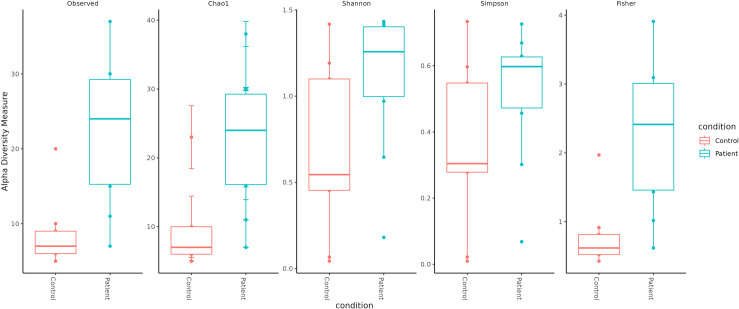
Alpha diversity analysis using different metrics in patients and controls.

**Fig 6:**
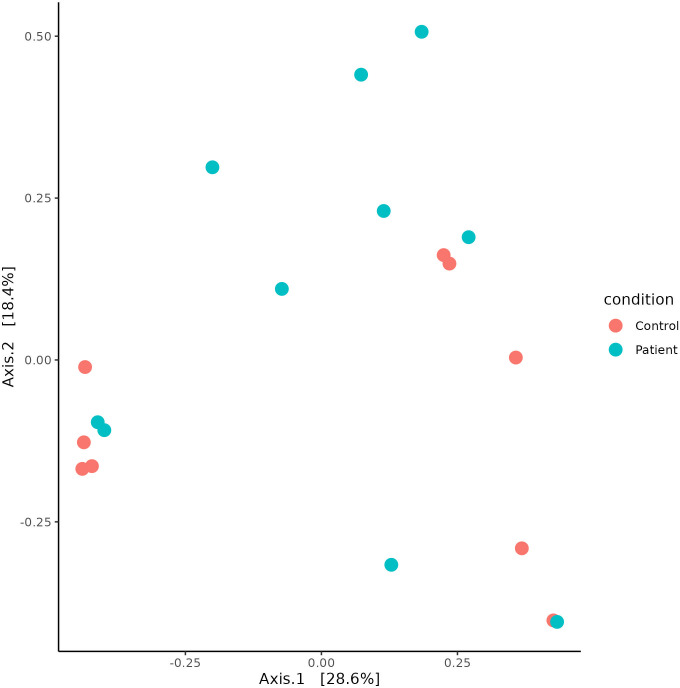
Microbial beta diversity analysis of patients vs controls.

**Fig 7:**
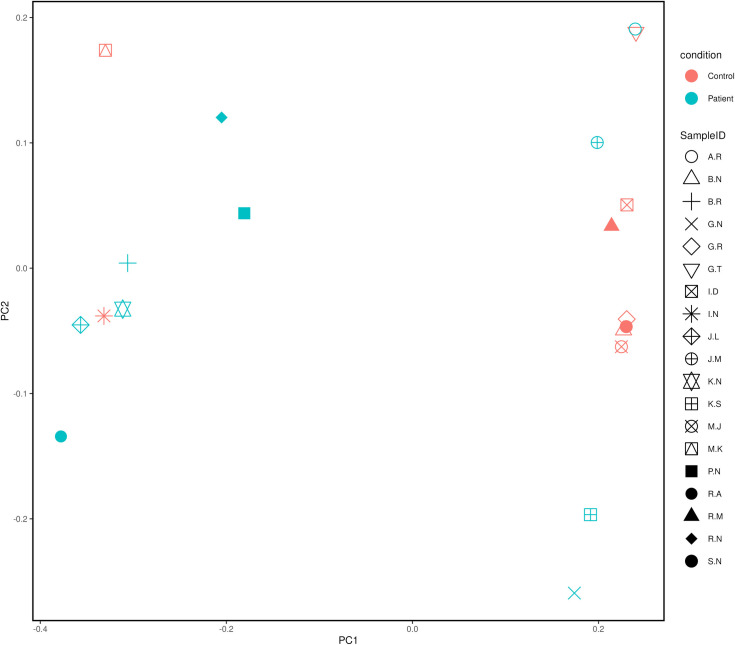
Principal component analysis showing comparison of microbial compositions in patients and controls.

Alpha diversity indices revealed that the patient group samples displayed higher diversity compared to the control group samples. The patient group exhibit higher diversity which is substantiated by the higher Shannon diversity index (Patient group: 1.10 ± 0.37, Control group: 0.66 ± 0.45, p value = 0.021) and Simpson diversity index (Patient group: 0.52 ± 0.18, Control group: 0.32 ± 0.23, p value = 0.028) compared to the controls. Both Shannon and Simpson diversity indices are significantly different (p < 0.05) indicating that patients exhibit greater microbial diversity than controls. These variations are supported by the boxplot (Fig 5), which shows a greater median diversity in both indices for the patient group. This indicates that each sample is unique and exhibits variations in diversity levels (Fig 5). Beta diversity analysis also revealed difference in bacterial composition between the patient and control samples (Fig 6); however, the p-value was not significant (P = 0.413). The principal component analysis plot also revealed that patient group samples were more diverse than the control group samples (Fig 7). The distance between sample points on the PCA plot reflects the similarity or dissimilarity of their microbiomes. Outlying samples in the PCA plot are those with atypical microbial compositions compared to most samples. The phylogenetic relationship of all OTUs across the patient and control groups is depicted in S3 Fig.

## Discussion

Studies carried out in recent years have highlighted the dysbiosis of the skin microbiome in different skin disorders [10,12,20–23]. In this line, the current study aimed to compare the skin microbiome profile of a neglected tropical disease, i.e., filarial lymphedema with that of healthy volunteers, employing a 16S rRNA based metagenomic approach. A notable difference in the skin microbiome profile between the two groups was observed. Both groups showed an abundance of phylum Firmicutes and Proteobacteria, while phylum Actinobacteria was found more frequently in the patient group. Remarkably, *Staphylococcus* emerged as the most prevalent genus in both groups, constituting 63% of the total genera in the patient group and 44% in the control group. The patient group’s diversity analysis, including alpha and beta diversity, revealed a higher diversity of bacteria. A significant finding is the detection of a rare bacterium *Eremococcus* in a few patients [23]. Certain other genera namely *Facklamia, Dermabacter, Acranobacterium, Terribacillus, Aerococcus, Macrococcus, Helcococcus, Idiomarina, Enhydrobacter, and Mannheimia* were detected in some patients but in none of the controls. The abundance and diversity of the skin bacterial flora in affected patients and the presence of emerging pathogens like *Eremococcus and Facklamia* may play a role in recurrent ADL attacks and disease progression. Presence of altered microbiome suggests that approaches for modulating the skin microbiome may be considered as a management strategy in the future.

To our knowledge, there are no published studies on the skin flora of patients with filarial lymphedema employing high-throughput approaches like 16S rRNA metagenomic technique. There is one report of a culture-based study from Ghana where MALDI TOF MS technique was used for identification of the isolated microorganisms [10]. This study from Ghana differs from the current study from India in many aspects. Samples were collected from sites with purulent discharge, wounds and also from skin of lymhedematous tissue [10]. Also, this study did not have a control arm with healthy individuals and therefore they compared the results with a study on skin microbiome profile of healthy individuals from the US [24]. Samples were taken longitudinally from patients with filarial lymphedema at three separate sampling sites and at six-month intervals. Microbes identified belonged to 4 major bacterial phyla: Firmicutes (69.79%), followed by Proteobacteria (16.57%), Actinobacteria (13.34%), and Bacteroidetes (0.29%). Species richness and diversity were higher in phylum Proteobacteria (22 genera), followed by Firmicutes (12 genera), Actinobacteria (8 genera), and Bacteroidetes (1 genera). Nearly 43 different bacterial genus were identified in this study. In our study, Firmicutes was the dominant phylum, followed by Proteobacteria and Actinobacteria. The phylum Bacteroidetes was not detected in the patient group. The phylum Firmicutes has 13 different genera, followed by Proteobacteria (6 genera), and Actinobacteria (5 genera). Remarkably, the bacterial flora such as *Staphylococcus, Micrococcus, Bacillus, Corynebacterium, Streptococcus, Enterococcus, klebsiella, Acinetobacter, Pseudomonas, Enterobacter, Escherichia, Macrococcus, Dermabacter, Lysinibacillus and Arcanobacterium* were the commonly seen bacterial genus among patients with filarial lymphedema in both the studies.

Notable changes in skin microbiome profile have been observed in other skin diseases as well and it would be worthwhile to compare our findings with these studies. Bayal et al. utilized next-generation 16S rDNA sequencing to compare the skin-associated microbial diversity of leprosy lesions with that of healthy volunteers. In patients with leprosy, there was an increased representation of *Pseudomonas, Methylobacterium,* followed by *Propionibacterium and Corynebacteriu*m. The genus *Staphylococcus* was less frequently observed in both affected and unaffected sites in patients with leprosy [12].This is a striking contrast with our findings in filarial lymphedema where *Staphylococcus* was dominant in patients.

A 16S rRNA metagenomic study conducted by Suwarsa et al. in Indonesia by collecting skin swabs from cubital fossa can help us understand the bacterial abundance of atopic dermatitis (AD) [20]. In mild AD, Firmicutes were the most abundant phylum, followed by Proteobacteria and Actinobacteria. Interestingly, in moderate AD patients, Proteobacteria was dominant, followed by Firmicutes and Actinobacteria. This study also identified *Staphylococcus* as the predominant genus among moderate AD patients, followed by *Ensifer and Bacillus*. This observation is similar to our findings among filarial lymphedema [20]. In another major skin disorder, psoriasis, Firmicutes was the most abundant and diverse phylum populating the psoriatic lesions and when compared to patient samples from uninvolved skin [21]. *Cutibacterium* species (formerly known as *Propionibacterium*) were less prevalent in lesion sites, but their levels were intermediate in unaffected skin regions of psoriatic patients. *Corynebacterium, Staphylococcus, Streptococcus,* and *Cutibacterium* were the most common genera found in both affected and normal skin of psoriasis patients and healthy individuals. *Streptococcus* was found more frequently in psoriatic lesion samples. In normal skin samples from patients and healthy individuals, the most common and diverse phylum, *Actinobacteria*, was substantially under-represented in the samples from psoriatic lesions [21].

Chun-xi et al. employed 16S rRNA gene sequencing to study the skin microbiome of patients with acne and healthy controls [22]. Four bacterial phyla (Actinobacteria,Firmicutes, Proteobacteria, and Bacteroidetes) dominated the skin microbiome of patients with acne and the relative abundance of the genera *Oscillospira, Enhydrobacter, and Bacteroides* was significantly higher in these patients. Bacterial composition and diversity were comparable among those with grade 1–3 acne. However, patients with grade 4 acne exhibited a distinct skin microbial profile, in contrast to those with grade 1–3 acne, characterised by an abundance of *Faecalibacterium, Klebsiella, Odoribacter, and Bacteroides*.

Among the healthy controls in our study, Firmicutes and Proteobacteria were the most prevalent phyla whereas Bacteroidetes and Actinobacteria were not detected. *Staphylococcus and Bacillus* are the major genera detected in healthy controls. Bayal et al also observed that the genus *Staphylococcus* was largely observed in control groups gathered from two distinct sites, which corroborates the findings in healthy controls in our study [12]. *Staphylococcus* was the most prevalent genus among healthy controls in the study by Suwarsa et al as well [20]. Grice et al. analysed the skin microbiota of 10 healthy individuals, revealing the presence of 16 phyla [24]. Among these, the most prevalent phyla were Actinobacteria (51.8%), Firmicutes (24.4%), Proteobacteria (16.5%), and Bacteroidetes (6.3%)*.* This study also documented the topographical and temporal diversity of the human skin microbiome.

In patients with filarial lymphedema, the skin and underlying tissues are particularly susceptible to secondary bacterial infections due to compromised immune function and disrupted skin integrity. While genera such as *Aerococcus, Facklamia,* and *Mannheimia* have not been found to be associated with ADL in individuals with filarial lymphedema, all these microorganisms have been recognized as primary infectious agents in other conditions, particularly as opportunistic pathogens. Consequently, if these organisms are part of the skin’s microbial flora, they may increase the risk of secondary bacterial infections or ADL in patients with filarial lymphedema as well [10,25]. However, this remains unknown as many of them are difficult to culture and only scant information is available in the literature. The genera *Facklamia* and *Mannheima* are reported to cause skin and soft tissue infections [26–28]. The current study throws light on the necessity to investigate these organisms in future studies and in relation with filarial lymphedema.

Though a consistent difference in the microbiome profile between patients and control groups as well as between different skin disorders is notable in different studies, the site of sample collection, the methodology applied for bacterial identification and the topographical differences in skin flora also need to be considered carefully for a better characterisation and description of the distinct microbiome in future works.

### Limitations

This study has certain limitations. Firstly, it was conducted with a small sample size, potentially affecting the generalizability of the findings. It might be impossible to define significant variations with a limited sample size. While we have carried out only one-time sample collection from patients and controls, the topographical and temporal diversity of the human skin microbiome is known, which we have not analysed in the current study. Another limitation is that we carried out the metagenomic analysis of samples from overnight cultures and direct sequencing of skin swab DNA samples was not performed. This will reflect the microbial community that grows under specific conditions. As a result, we might have overlooked certain anaerobic and unculturable bacteria.

## Conclusion

The current metagenomic study on the skin microbiome of patients with filarial lymphedema revealed a notable difference in the bacterial flora between patients and healthy controls. Future studies need to be carried out in different settings with a larger number of samples in different stages of the disease and at multiple time points for a better characterisation and also should focus on understanding the role of skin microbiome in recurrent ADL attacks and lymphedema progression.

## Supporting information

S1 Fig Heat tree analysis of bacterial communities in the patient group. (TIF)

S2 Fig Heatmap of the most abundant genera identified in patient and control groups. (TIF)

S3 Fig Bacterial genus-based phylogeny showing abundance among lymphedema patients vs healthy controls. (TIF)
