# Supplementary figures and images for: Comparative analysis of skin microbiome of patients with filarial lymphedema and healthy individuals
Source: PLoS One. 2025 Jul 2;20(7):e0325380. doi: 10.1371/journal.pone.0325380 (PMC12221001; doi:10.1371/journal.pone.0325380)

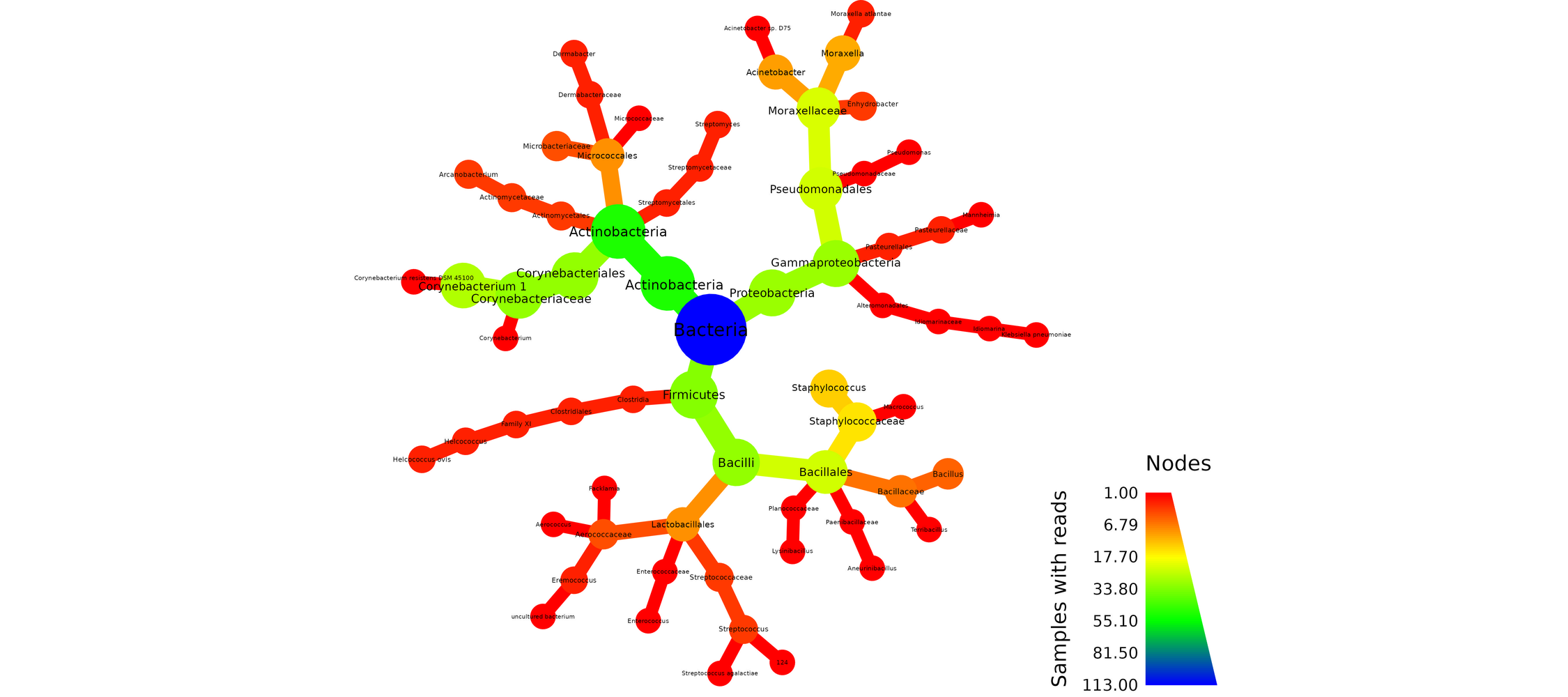

Supplement: S1 Fig — (TIF) [file pone.0325380.s001.tif]

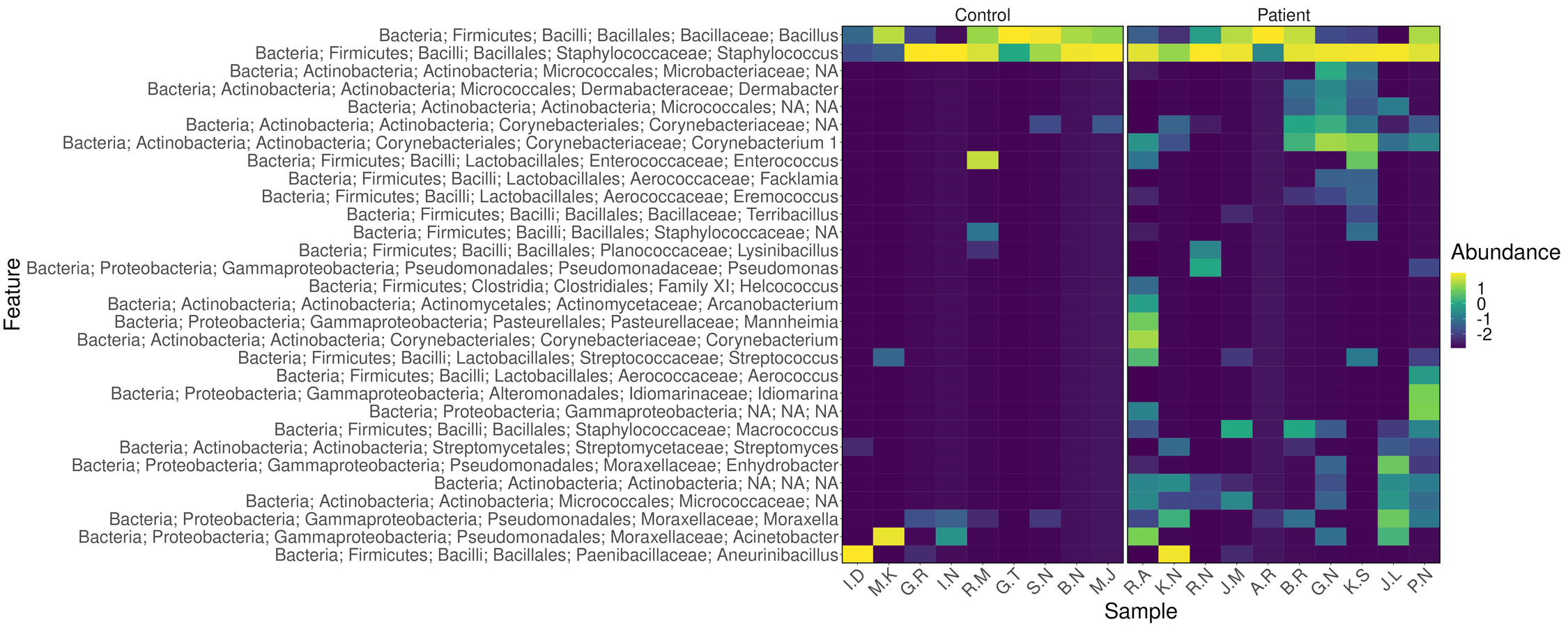

Supplement: S2 Fig — (TIF) [file pone.0325380.s002.tif]

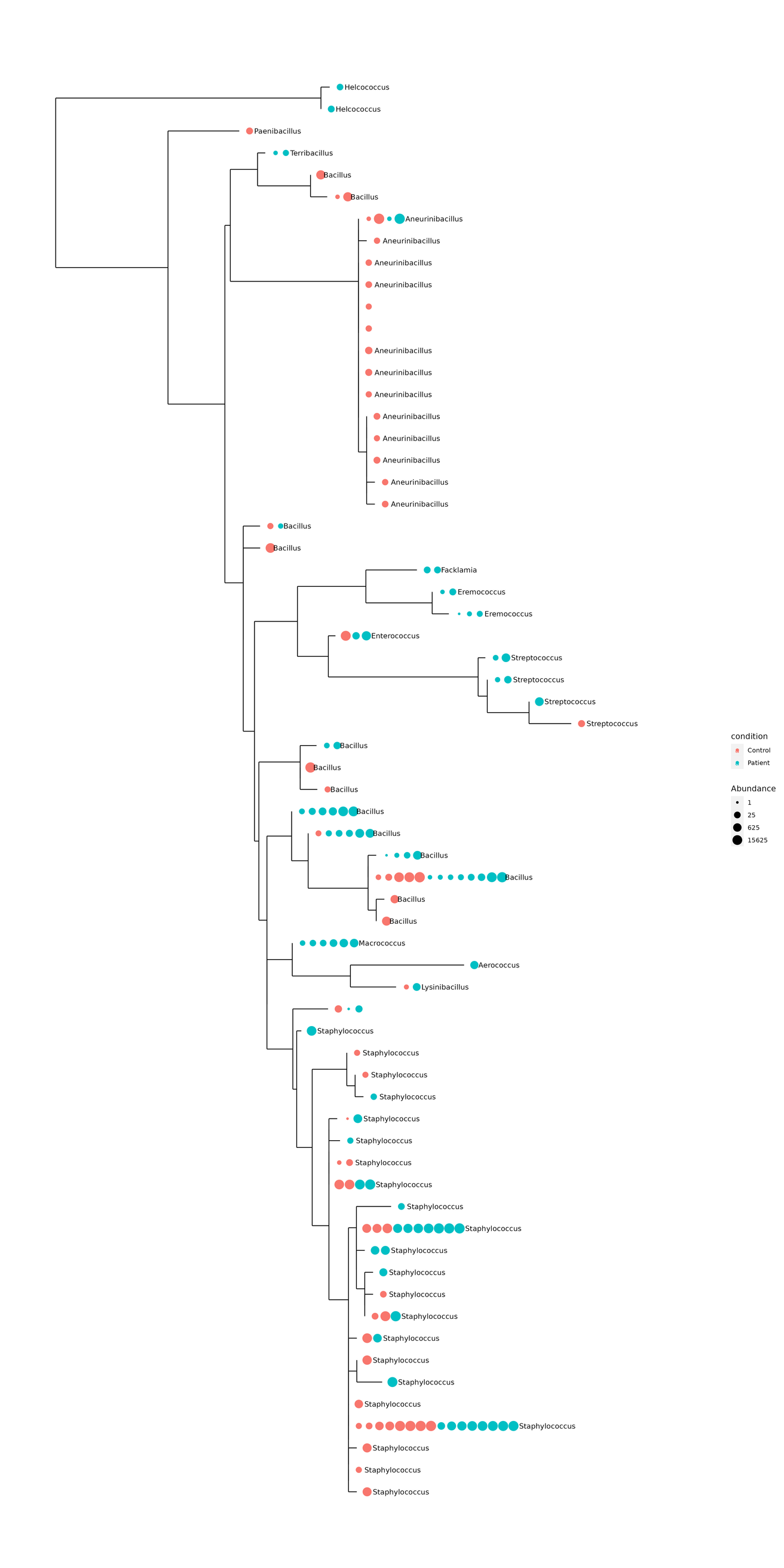

Supplement: S3 Fig — (TIF) [file pone.0325380.s003.tif]
